# Lacertus syndrome: a commonly missed and misdiagnosed median nerve entrapment syndrome

**DOI:** 10.1186/1753-6561-9-S3-A74

**Published:** 2015-05-19

**Authors:** Donald Lalonde

**Affiliations:** 1 Department of Plastic and Reconstructive Surgery, Dalhousie University, Saint John, New Brunswick, Canada, E2K 1J5

## 

Carpal tunnel syndrome is a compression of the median nerve which occurs under a sheet of ligamentous tissue (transverse carpal ligament) just past the wrist joint. Lacertus tunnel syndrome is a compression of the median nerve which occurs under a sheet of ligamentous tissue (lacertus fibrosus) just past the elbow joint. Lacertus syndrome has been well described by Hagert (*Hagert E. Clinical diagnosis and wide-awake surgical treatment of proximal median nerve entrapment at the elbow: a prospective study. Hand (NY). 2013; 8(1): 41-6.)*

The most common complaints in patients with lacertus syndrome are a loss of key and tip pinch strength, a loss of fine motor skills and sense of clumsiness (dropping objects), and, rarely, transient paresthesias in the median nerve innervated region of the hand (thumb-radial aspect of ring finger. They also may get burning and numbness in the palmar cutaneous branch of the median nerve distribution. The patients will usually exhibit: (1) Distinct weakness when manually testing the strength of the muscles innervated by the median nerve distal to the lacertus fibrosus, especially the FPL, FDP II, and FCR. (2) External pressure of the median nerve at the level of the lacertus fibrosus will elicit distinct pain and, at times, a positive Tinel’s sign.

This proximal median nerve compression can coexist with carpal tunnel syndrome. If someone is still having symptoms after carpal tunnel release, the hand should be examined for lacertus syndrome. A decrease of power of FPL, FDP2, and FCR as well as tenderness at the medial edge of the lacertus fibrosus over the median nerve will make the diagnosis. Nerve conduction studies are not helpful.

The surgery is performed with the patient awake and no tourniquet. After injection of 30cc of 1% lidocaine with 1:100,000 epinephrine and 3cc of 8.4% bicarbonate to decrease the acidic sting of the local anesthetic, 30 minutes are allowed to elapse to let the epinephrine work. The patient can be injected in the waiting area before entering the operating room.

A 2–3-cm transverse incision is placed in the flexion crease of the cubital fossa, from 1 cm medial of the biceps tendon to 2 cm lateral of the medial epicondyle. Careful dissection is made subcutaneously to the pronator teres fascia, taking great care to identify and protect branches of the medial antebrachial cutaneous nerve. The pronator teres fascia is incised and followed laterally, allowing exposure of the lacertus fibrosus, which is subsequently divided. By retracting the pronator teres muscle medially, the median nerve is readily exposed. Any focal adherences to the underlying brachialis muscle may then be released. At this point, the strength of the FPL and FDP II is again tested intraoperatively before the skin is closed, as return of muscle strength is usually immediate after proper release of the nerve.

After cauterization, the wound is closed with buried interrupted sutures, a small soft dressing applied and immediate mobilization encouraged. Patients with no manual labor return to work within 1–2 days postoperatively.

Many patients with residual symptoms after carpal tunnel release will get better after lacertus release.

